# Finnish Parkinson’s disease study integrating protein-protein interaction network data with exome sequencing analysis

**DOI:** 10.1038/s41598-019-55479-y

**Published:** 2019-12-11

**Authors:** Ari Siitonen, Laura Kytövuori, Mike A. Nalls, Raphael Gibbs, Dena G. Hernandez, Pauli Ylikotila, Markku Peltonen, Andrew B. Singleton, Kari Majamaa

**Affiliations:** 1 Research Unit of Clinical Neuroscience, University of Oulu, Oulu, Finland; 2 Department of Neurology and Medical Research Center Oulu, Oulu University Hospital and University of Oulu, Oulu, Finland; 3 Laboratory for Neurogenetics, National Institute on Aging, National Institutes of Health, Bethesda, MD USA; 4 Data Tecnica International, Glen Echo, MD 20812 USA; 5 Institute of Clinical Medicine, Department of Neurology, University of Turku, Turku, Finland; 6 Division of Clinical Neurosciences, Turku University Hospital, Turku, Finland; 7 THL, Helsinki, Finland

**Keywords:** Proteome informatics, Next-generation sequencing, Biochemical networks, Parkinson's disease

## Abstract

Variants associated with Parkinson’s disease (PD) have generally a small effect size and, therefore, large sample sizes or targeted analyses are required to detect significant associations in a whole exome sequencing (WES) study. Here, we used protein-protein interaction (PPI) information on 36 genes with established or suggested associations with PD to target the analysis of the WES data. We performed an association analysis on WES data from 439 Finnish PD subjects and 855 controls, and included a Finnish population cohort as the replication dataset with 60 PD subjects and 8214 controls. Single variant association (SVA) test in the discovery dataset yielded 11 candidate variants in seven genes, but the associations were not significant in the replication cohort after correction for multiple testing. Polygenic risk score using variants rs2230288 and rs2291312, however, was associated to PD with odds ratio of 2.7 (95% confidence interval 1.4–5.2; p < 2.56e-03). Furthermore, an analysis of the PPI network revealed enriched clusters of biological processes among established and candidate genes, and these functional networks were visualized in the study. We identified novel candidate variants for PD using a gene prioritization based on PPI information, and described why these variants may be involved in the pathogenesis of PD.

## Introduction

The genetic etiology of Parkinson’s disease (PD) is complex (see e.g.^1^). Many variants are associated with PD, but the effect of each variant seems to be small^2^. Hence, large sample sizes will be required in a successful search for new variants^3^, and the probability of success can be further increased by employing information on genes possibly associated with the disease. Reduction of variants in the analysis can be accomplished by combining protein-protein interaction (PPI) data with genomic data (see e.g.^4^).

We have previously conducted a whole exome sequencing (WES) study on Finnish PD patients and population controls^5^. Here, we carried out a single variant re-analysis of these subjects and included an additional replication cohort of Finnish ancestry in the analysis. PPI information enabled us to focus the analysis on 36 genes with established or suggested associations with PD (PD36) and their interaction partners. Single variant analysis (SVA) in the discovery dataset yielded 11 candidate variants in seven genes, which were then analyzed in the replication dataset. Polygenic risk score (PRS) was calculated with two of the candidate variants and association to PD was tested in the replication dataset. In order to visualize possible biological processes related to these genes, we created PPI networks that included functional information of PD36 genes and functional information of novel candidate genes and genes of PD associated loci^6,7^.

## Subjects and Methods

### Study populations and WES data preparation

Details of the three Finnish studies, Mitopark, Stampeed and FINRISK, have been described previously^5,8^. Exome sequencing of the FINRISK-study has been performed at McDonnell Genome Institute, Washington University, and variant calling at Broad Institute as described previously^9^ and summarized in Supplementary Material.

Quality control of the exome sequences in Mitopark, Stampeed and FINRISK datasets was carried out with methodology described previously^5^. The discovery dataset (men 46%) consisted of exome sequences from Mitopark (N = 392 cases), Stampeed (N = 493 controls) and FINRISK (N = 47 cases; N = 362 controls). Total genotyping rate was 0.98. A portion of FINRISK cases (N = 107) were randomly assigned to the discovery and replication datasets, and in the discovery dataset the FINRISK controls were matched to cases with respect to sex and age. Only variants found in all the three studies were included in the merged discovery dataset. The replication dataset (men 48%) consisted of 60 FINRISK cases and 8214 FINRISK controls.

The study was approved by the Ethics Committee of the Turku University Hospital. All the methods were carried out in accordance with the relevant guidelines and regulations and informed consent was obtained from all participants.

### Gene prioritization using protein-protein interaction data

UniProt database (version 2018-02) was queried with the phrase *parkinson disease:disease AND organism*:“*Homo sapiens (Human) [9606]”* in order to find proteins related to PD. In total, 36 proteins (Table 1) with established or suggested associations with Parkinson’s disease (PD36 proteins) were found. Information on protein-protein interactions was downloaded from Integrated interactions database (IID)^10^. Experimentally detected human specific interactions (version 2017-04) contained 18,627 vertices and 280,845 interactions. We then created a network (Supplementary Fig. S1) that included PD36 proteins and proteins that have direct interactions with these proteins. In total, the network (PD2300net) consisted of 2305 UniProt protein identifiers.

**Table 1:** Genes with suggested associations with Parkinson’s disease that were used to build PD2300net.

#	Uniprot ID	Gene Symbol	#	Uniprot ID	Gene Symbol
1	Q9NQ11	ATP13A2	19	P49821	NDUFV1
2	Q9Y6H1	CHCHD2	20	Q99497	PARK7
3	O75165	DNAJC13	21	O95263	PDE8B
4	O75061	DNAJC6	22	Q9BXM7	PINK1
5	Q04637	EIF4G1	23	O60733	PLA2G6
6	Q9Y3I1	FBXO7	24	P54098	POLG
7	P04062	GBA	25	Q9UGJ0	PRKAG2
8	Q6Y7W6	GIGYF2	26	O60260	PRKN
9	O43464	HTRA2	27	P37840	SNCA
10	Q5S007	LRRK2	28	Q9Y6H5	SNCAIP
11	P10636	MAPT	29	Q13501	SQSTM1
12	P03886	MT-ND1	30	O43426	SYNJ1
13	P03897	MT-ND3	31	Q9BSA9	TMEM175
14	P03915	MT-ND5	32	Q96A57	TMEM230
15	Q8N183	NDUFAF2	33	P09936	UCHL1
16	Q5TEU4	NDUFAF5	34	P55072	VCP
17	O43181	NDUFS4	35	Q709C8	VPS13C
18	O75251	NDUFS7	36	Q96QK1	VPS35

### Whole exome sequencing data analysis

**Figure 1:**
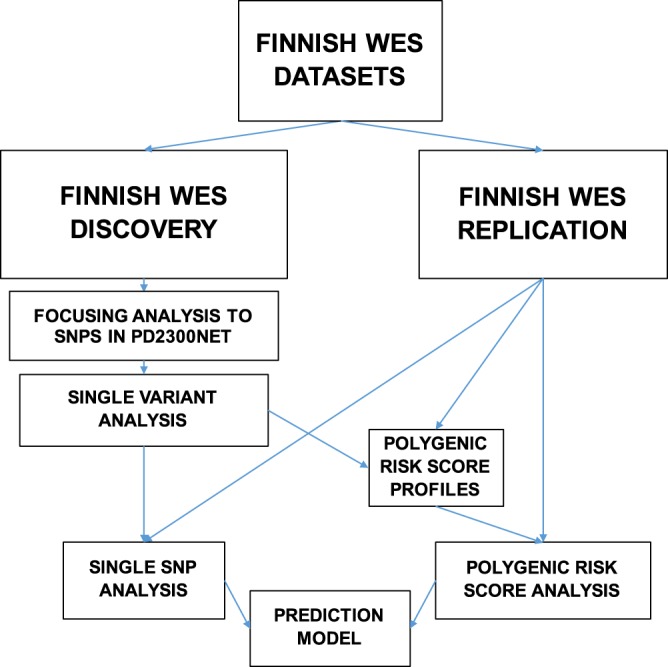
Whole exome sequencing data analysis workflow.

Analysis workflow is shown in Fig. 1. Whole exome sequences (WES) from the three studies were subjected to single-variant association (SVA) test and polygenic risk score (PRS) association test. Analysis was focused to 36 genes with established or suggested associations with PD (PD36) and 2269 genes that interacted with them in PD2300net. The discovery dataset was filtered to include only variants in genes in PD2300net and variants 20 kbp upstream or downstream of PD2300net genes. In total, there were 8091 variants in the discovery set.

SVA test and PRS association test were performed using logistic regression with the first ten principal components as covariates. Genomic inflation factor lambda (based on median chi-squared test) was 1.0 in the discovery dataset. Variants with empirically set p value threshold of 0.0005 (N = 11) were used in logistic regression analysis in replication dataset, using the same settings as in the discovery dataset except that age was added into the covariates. Bonferroni correction for multiple testing was applied to replication results. Sanger sequencing of samples in the discovery dataset confirmed that variants rs113574896 and chr10_105048270_AGAG_A were false positive findings. Variants rs2627037, rs922984, rs2291310, rs2291311, rs2291312 in TTN gene were in linkage disequilibrium (LD) with each other and had almost identical frequency profiles (Table 2).

**Table 2:** Single variants in the discovery and replication datasets.

Discovery Set											Replication Set					
GENE	SNP	CHR	BP	A1	OR	P	C_A	C_U	F_A	F_U	OR	P	C_A	C_U	F_A	F_U
UBXN11	rs117509001	1	26629342	A	5.979	0.0004805	13	6	0.0148	0.0035	NA	NA	0	80	0	0.00487
GBA	rs2230288	1	155206167	T	2.208	8.927e-06	74	81	0.0855	0.0474	2.137	0.02379	10	676	0.083	0.04115
TTN	rs2627037	2	179606538	A	1.616	0.0004346	121	166	0.1378	0.0971	1.61	0.05265	20	1780	0.167	0.1084
TTN	rs922984	2	179615887	T	1.637	0.0003337	119	163	0.1355	0.0953	1.64	0.04411	20	1749	0.167	0.1065
TTN	rs2291310	2	179623758	C	1.637	0.0003337	119	163	0.1355	0.0953	1.642	0.04356	20	1747	0.167	0.1063
TTN	rs2291311	2	179629461	C	1.637	0.0003337	119	163	0.1355	0.0953	1.641	0.04386	20	1748	0.167	0.1064
TTN	rs2291312	2	179631214	C	1.637	0.0003337	119	163	0.1355	0.0953	1.64	0.0441	20	1749	0.167	0.1065
IKBKB	rs140485496	8	42178280	T	2.666	0.0001978	31	34	0.0353	0.0199	NA	NA	0	376	0	0.02289
MIR7705/PABPC1	rs113574896	8	101717195	C	3.987	1.122e-11	84	46	0.0966	0.0269	NA	NA	0	10	0	0.000609
INA	chr10_105048270_AGAG_A	10	105048270	A	5.722	5.064e-05	14	11	0.0294	0.0064	2.782	0.01913	6	302	0.05	0.01838
KARS/TERF2IP	rs1865493	16	75681743	G	0.548	5.161e-05	84	242	0.0957	0.1417	1.169	0.54	18	2130	0.15	0.1297

Discovery set: cases N = 439; controls N = 855; replication set: cases N = 60; controls N = 8214; Bonferroni cutoff p < 0.0045; OR = odds ratio; C_A = Allele 1 count among cases; C_U = Allele 1 count among controls; F_A = Allele 1 frequency among cases; F_U = Allele 1 frequency among controls.

Polygenic risk score was calculated using variants rs2230288 and rs2291312. These variants were selected on the basis of p-value cutoff 0.0005 in the SVA test and it was required that minor allele frequency was similar in the discovery and replication datasets. False positive variants rs113574896 and chr10_105048270_AGAG_A were removed from the analysis. Furthermore, all the combinations of the five variants in LD in TTN gene yielded similar PRS results and therefore variant rs2291312 was included to represent the LD block. Selected variants rs2230288 and rs2291312 were weighted by logarithm of odds ratio, and these values were added together for each sample in order to obtain PRS.

For disease prediction, the discovery dataset was used in training and the replication dataset in testing the model. Separate models were built on the two variants as features (variant model) and the polygenic risk score as the feature (PRS model). Variant model used random forest and PRS model logistic regression as classifier. The models were evaluated with the aid of sensitivity, specificity, area under curve (AUC), balanced accuracy score and mean decrease in impurity as the main metrics^11,12^ (MDI). Balanced accuracy score is equal to the arithmetic mean of sensitivity and specificity.

Depth of coverage was analysed in a random sample of 400 cases or controls from the discovery dataset.

### Visualization of protein-protein interaction network

**Figure 2:**
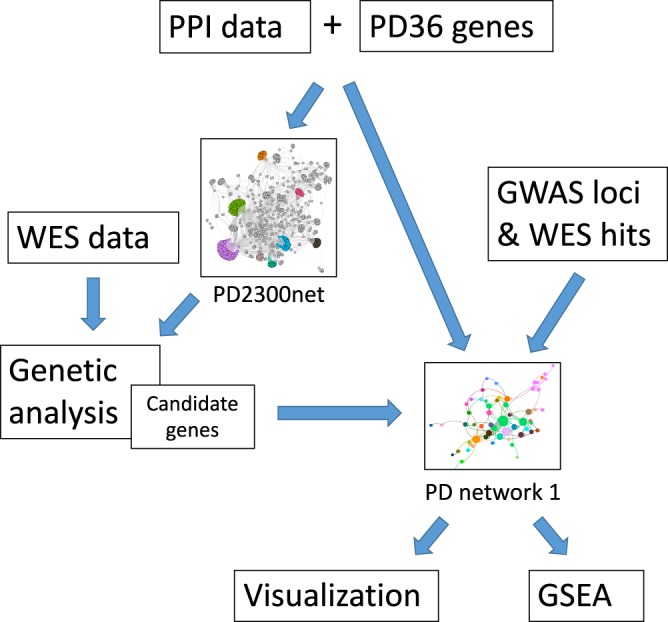
Workflow of creating visualization of protein-protein interaction network. PPI, protein-protein interaction; WES, whole exome sequencing; GWAS, genome-wide association study; GSEA, gene set enrichment analysis.

Visualization network was built using experimentally detected human-specific interactions of the IID database. Interactors in this network were PD36 proteins, corresponding proteins of the candidate genes, and corresponding proteins of the genes from two genome-wide association study (GWAS) meta-analysis studies and from our previous WES and GWAS study^5–7^ (Fig. 2). In order to select genes related to loci in GWAS studies, genes were retrieved within 250kbp up- and downstream of the reported chromosome positions (Supplementary Fig. S2). Genes that belonged to PD2300net were selected and the original chromosome positions of these genes (GWAS hits) are shown in Supplementary Table S1-S3. Furthermore, genes that belonged to PD2300net and were either significant in our previous WES study in gene-level analysis or contained de-novo variants with high effect size (Supplementary Table S4).

**Figure 3:**
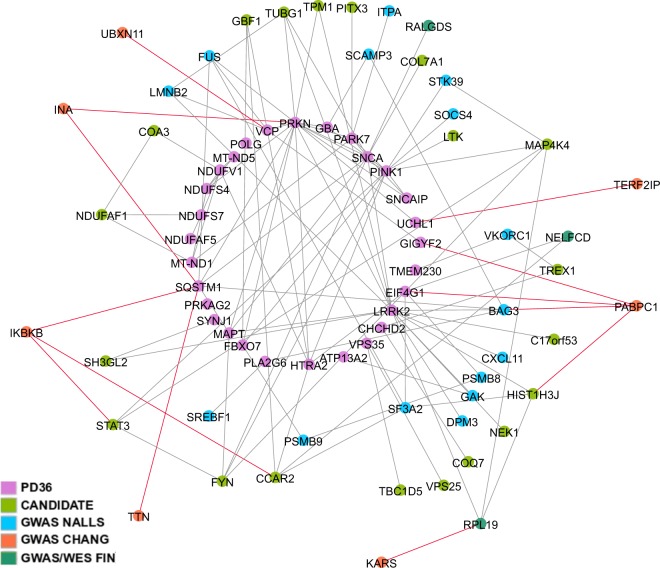
PD network 1. Protein-protein interaction network visualizing the interactions between established and suggested PD genes and candidate genes. Interactions (edges) of the seven novel candidate genes are highlighted in red color. Abbreviations: PD36, 36 established or suggested PD genes; CANDIDATE, seven novel candidate genes; GWAS NALLS, GWAS hits in Nalls *et al*.^7^ meta-analysis discovery phase; GWAS CHANG, GWAS hits in Chang *et al*.^6^ meta-analysis discovery phase; GWAS/WES FIN, significant GWAS hits and selected WES hits in Siitonen *et al*.^5^.

The visualization network (PD network 1) included edges between PD36 proteins, GWAS or WES hits and candidate proteins. This network of 74 proteins incorporated 29 PD36 proteins, 38 GWAS or WES hits and seven novel candidate protein hits with 165 edges (Fig. 3, Supplementary Table S5). The largest connected component of PD network 1 consisted of 64 genes.

Randomization test was performed on PD network 1. The main metrics were average path length (APL) and average clustering coefficient (ACC). Random graphs (N = 10,000) with size and degree distribution similar to PD network 1 were generated from IID database data by label permutation, and one-sided Monte Carlo p value was calculated for the true APL and ACC values. The degree distributions of the random graphs were preserved by binning proteins into 30 equal sized bins by their network degree distribution and swapping the protein labels between the proteins in the same degree bin without replacement.

### Programs and databases

Plink 1.9b^13^ and R version 3.4.3 were used to prepare and analyze the exome sequences. Python version 3.5 with Scikit-learn library version 0.19.2 was used for logistic regression and random forest prediction models and MDI analysis^14^. Linkage disequilibrium between TTN variants was tested with LDlink webtool setting Europeans as the population^15^. Depth of coverage analysis was conducted using Genome Analysis Toolkit version 3.1^16^.

All networks were built and visualized using Python 3.5, Pyensembl version 1.1.0, Spark version 2.1.0, Graphframes version 0.5.0, Python library NetworkX version 2.1 and Gephi version 0.9.2. Random graphs for PPI network randomization test were built using in-house scripts. Average path length and average clustering coefficient was calculated using Stanford Network Analysis Platform (SNAP) version 5.0^17^. Pathway and gene enrichment information was acquired from GSEA v6.1 (software.broadinstitute.org/gsea) and STRING v10^18^ databases.

## Results

### Single-variant association test

We identified 8091 variants in PD2300net genes among 439 cases and 855 controls in the discovery dataset. Several p value thresholds from 0.05 to 0.00005 were tested and the lowest threshold 0.0005 that identified more than ten variants was selected for further evaluation in order to keep the false positive rate low. Eleven variants with p value less than 0.0005 in six novel genes and one established risk gene (candidate genes) were selected for replication, but none of the associations was significant in the replication dataset after correction for multiple testing (Table 2). One of the 11 variants was rs2230288 in the *GBA* gene leading to p.E365K (legacy name p.E326K). The frequency of this allele was 8.5% in PD cases and 4.7% in controls in the discovery dataset and similar frequencies were observed in the replication dataset (Table 2) giving an odds ratio of 2.1. The association of the variant with PD was not significant after correction for multiple testing, however.

We estimated the depth of coverage in discovery dataset. At the whole exome level 90% of all the contigs in our study were covered at 20x depth. However, only 19% of the PD36 genes were covered at 20×, with average total coverage being about 14x for these genes.

### Polygenic risk score based on two variants is associated with the risk of PD

The variants rs2230288 and rs2291312 that passed the selection criteria were included in the calculation of polygenic risk score (PRS). The mean of PRS was 0.17 (range; 0.00 to 1.78) and 18% of the cases and 9% of the controls belonged to the upper quartile of the PRS values. The association of PRS with PD was tested in the replication dataset giving a mean odds ratio of 2.7 (95% confidence interval 1.4–5.2; p < 2.56e-03) (Table 3).

**Table 3:** Logistic regression results of polygenic risk score in the replication dataset.

	P	FDR	Bonf	OR	2.5%	97.5%	Estimate	std.error	Statistic
PRS	2.56e-03	1.19e-02	3.58e-02	2.7078	1.4175	5.1728	1.00	0.33	3.016
AGE	5.26e-07	3.68e-06	7.37e-06	1.0584	1.0352	1.0821	0.06	0.01	5.016
PC1	4.16e-02	1.46e-01	5.83e-01	0	0	0.3028	−31.55	15.49	−2.037

PRS = Polygenic risk score; AGE = age at onset/age at sampling; PC1 = principal component 1; P = p value; FDR = False discovery rate; Bonf = Bonferroni correction; OR = Odds ratio; 2.5% = 95% lower confidence; 97.5% = 95% upper confidence.

Two prediction models (variant model, PRS model) were then trained with the discovery dataset and tested in the replication dataset. Predictive power of the two models was generally low (Tables 4 and 5). Balanced accuracy score was 0.56 and the area under curve (AUC) score was 57%. The models classified 27% of the controls and 38% of the cases as cases.

**Table 4:** Metrics of prediction models in the replication dataset.

Model	Accuracy	Specificity	Sensitivity	Bal. accuracy	AUC	95%CI
PRS	0.73	0.73	0.38	0.56	0.57	0.499–0.65
Variant	0.73	0.73	0.38	0.56	0.57	0.501–0.63

PRS = Polygenic risk score model; Variant = Variant model; Bal. accuracy = Balanced accuracy score; AUC = area under curve score; 95%CI = AUC 95% confidence interval.

**Table 5:** Confusion matrix of models in the replication dataset.

	Predicted as cases	Predicted as controls
Actual Cases	23 (TP)	37 (FN)
Actual Controls	2199 (FP)	6015 (TN)

TP = True positive; FN = False negative; FP = False positive; TN = True negative.

### Analysis of the protein-protein interaction network

The protein-protein interaction network (PD network 1) included 29 of the PD36 proteins, 38 GWAS hits and seven novel candidate proteins with 165 edges (Fig. 3, Supplementary Table S5). The largest connected component of PD network 1 consisted of 64 genes.

Protein-protein interactions in PD network 1 revealed that the proteins encoded by the seven novel candidate genes interacted directly with seven PD36 genes and five GWAS or WES hits (Fig. 3). Furthermore, RALGDS was identified in PD network 1 as a possible source of the GWAS hit in locus chr9:135955826 (rs11243993), identified in our previous study^5^. Variant rs11243993 was identified in 14 cases and none of the controls in GWAS analysis, but WES analysis did not identify any significant RALGDS variants.

Randomization test was performed to PD network 1 in order to estimate the statistical significance of the created PPI network. The largest connected component had significantly shorter average path length (p < 0.01066) and significantly greater average clustering coefficient (p < 0.000414) than what was expected of random network with the same size and node degree distribution (Supplementary Fig. S3). This suggests that PD Network 1 could be considered as relatively small world in comparison to random graph.

Gene set enrichment analysis (GSEA) revealed that 28 proteins in PD network 1 were involved in phosphate metabolism (p < 5.72e-8), 29 proteins in phosphorylation (p < 1.62E-15) and 20 proteins in organonitrogen compound metabolism (p < 5.03E-11) (Supplementary Table S6).

An interaction between the established PD proteins and candidate proteins in PD network 1 was also evident on inspection of the information on cell signalling by protein phosphorylation (uniprot.org) (Supplementary Fig. S4). Similarly, we observed a functional network, where candidate proteins interact with the rest of the network, if we merged information on previously suggested biological processes in PD (such as ubiquitination, mitochondrial function, signaling cascades, transportation and RNA processing) into PD network 1 (Supplementary Fig. S5).

## Discussion

We analyzed here our previous WES data now focusing on a targeted set of 2305 genes. We compared 439 Finnish PD cases and 855 Finnish controls in the discovery phase and replicated the results in another dataset consisting of subjects with Finnish ancestry. SVA test was not significant in the replication dataset, but an association was found between PD and PRS. In addition, protein-protein interaction network showed that the novel variants identified here, loci identified in a recent GWAS meta-analysis^6,7^, selected significant hits in our previous GWAS and WES study^5^, and known PD genes formed a network with clusters of biological processes, further suggesting the involvement of these proteins in PD.

We found a significant association between PRS and PD in the replication dataset with an odds ratio of 2.7. Predictive PRS model was able to capture 38% of the cases, although with a high rate of false negative cases. The prediction rate was generally low both in the PRS model and in the variant model. The relative effect of the p.E365K variant in GBA in the classification was higher than that of TTN in the variant model with mean decrease in impurity (MDI) of 58%, leaving MDI of 42% to the TTN variant.

The clinical significance of the GBA variant p.E365K has been controversial (see Clinvar: RCV000487503), but a recent meta-analysis provided some evidence that p.E365K may indeed be associated with PD^19^. The association was not significant in our study after correction for multiple testing, but ten out of 60 patients carried this mutation in the replication dataset giving an allele frequency of 0.08, which was similar to that in the discovery dataset. Interestingly, the allele frequency of the variant in non-Finnish European populations is 0.01 according to gnomAD^20^, whereas we found a frequency of 0.04 in the Finnish population. Without genome-wide significant (p < 5e-8) results, our study cannot completely define the role of rs2230288 in PD.

Five variants (rs2627037, rs922984, rs2291310, rs2291311, rs2291312) were located in the titin (TTN) gene. TTN is a large gene, rich in variants and, therefore, it is possible that the association reflects variant ascertainment or sequencing bias. The Clinvar database reports 11,148 TTN variants, among which there are at least 251 pathogenic variants in 17 different conditions including cardiomyopathies, skeletal muscle phenotypes and congenital diseases^21^.

Titin acts in sarcomere assembly and has role in elasticity and resting tension of striated muscles^22,23^. The variants identified in our study were located in or in close proximity of immunoglobulin-like domains 19 and 20 that account for the elasticity of titin. In addition, oxidation of the domains has been shown to lead to stiffening of the protein^24^. Interestingly, the molecular spring titin determines, at least in part, muscle stiffness and rigidity and tremor are the clinical hallmarks in PD^25,26^. Muscle stiffness likely plays a role in determining the frequency of oscillatory motion and therefore the changes in titin structure could impact whole-animal movement by modulating muscle stiffness.

Only 19% of the PD36 genes in WES data had a depth of coverage of 20×. This may have caused a loss of significant findings in established PD genes and should be taken into consideration when estimating the results.

We composed a PPI network that was based on 36 PD genes. The network enabled us to identify genes that interact, in addition to the established PD genes, with genes in the vicinity of GWAS hits reported in previous meta-analyses, genes in the vicinity of significant GWAS hits in our previous study and selected WES hits from our previous study. Not all plausible PD genes were used to build the initial gene set and although this approach may reduce the number of candidate genes identified, the small initial set of PD genes should have reduced the number of false positive findings. PPI network visualizations and GSEA described the network context around the identified candidate genes and supported their relation to established PD genes. Furthermore, a review of the literature on the seven candidate genes indicated that they may be involved in neurodegenerative diseases (Supplementary Table S7, Supplementary Material). Interestingly, 64 proteins in PD network 1 were connected via interaction suggesting a linking factor between them or a common signaling cascade. Phosphate metabolism and phosphorylation were among the most common processes identified in the GSEA analysis, but also other previously known biological processes, such as mitochondrial processing, ubiquitination and response to stress were identified.

GWAS hit at locus rs11243993 from our previous study was suggested to originate from RALGDS gene in PD network 1. RALGDS has a role in GTPase regulation and in PD network 1 the protein interacts directly with SNCA^27^. Interestingly, GTPase signaling have been suggested to be the link between genomics and etiology of PD^28^.

PPI networks were built using experimental subset of IID, which integrates data from primary data sources, such as BioGRID or IntAct. These datasets use various experimental techniques as the source and each have its own strengths and pitfalls. Here we did not filter the PPI data for interaction confidence or characterization score, but instead expected the genetic association test to serve as evidence for plausible interactions.

We identified novel candidate variants in PD using a combination of WES data and PPI network data. Targeted gene analysis, polygenic risk score association analysis and PPI network analysis indicated that these variants may be involved in the pathogenesis of PD. The power in our study was limited, and therefore, our findings can be seen as hypothesis generating and they require further investigation.

## Supplementary information

Supplementary Information

## Data Availability

The datasets generated during and/or analysed during the current study are available from the corresponding author on reasonable request.
