# Supplementary material for: Finnish Parkinson’s disease study integrating protein-protein interaction network data with exome sequencing analysis
Source: Sci Rep. 2019 Dec 11;9:18865. doi: 10.1038/s41598-019-55479-y (PMC6906405; doi:10.1038/s41598-019-55479-y)
Supplement: Supplementary file 1 — Supplementary Information [file 41598_2019_55479_MOESM1_ESM.pdf]

Supplementary Information

Finnish Parkinson's disease study integrating protein-protein interaction network data with exome sequencing analysis

Ari Siitonen [a,b], Laura Kytövuori [a,b], Michael Nalls [c,d], Raphael Gibbs [c], Dena G. Hernandez [c], Pauli Ylikotila [e,f], Markku Peltonen [g], Andrew Singleton [c], Kari Majamaa [a,b]

Affiliations

[a] Institute of Clinical Medicine, Department of Neurology, University of Oulu, Oulu, Finland

[b] Department of Neurology and Medical Research Center, Oulu University Hospital, Oulu, Finland

[c] Laboratory for Neurogenetics, National Institute on Aging, National Institutes of Health, Bethesda, MD, USA

[d] Data Tecnica International, Glen Echo, MD, 20812 USA

[e] Institute of Clinical Medicine, Department of Neurology, University of Turku, Turku, Finland

[f] Division of Clinical Neurosciences, Turku University Hospital, Turku, Finland

[g] THL, Helsinki, Finland

Corresponding author

Ari Siitonen

Institute of Clinical Medicine, Department of Neurology, University of Oulu, Oulu, Finland

Department of Neurology and Medical Research Center, Oulu University Hospital, Oulu, Finland

Address: University of Oulu, Department of Neurology, P.O. Box 5000, FIN-90014 Oulu, Finland.

Email: Ari.Siitonen@iki.fi

Table of Contents

Acknowledgments 3

FINRISK methods 4

FINRISK WES laboratory methods and data preparation 4
Library Construction 4
In-solution hybrid selection for exome enrichment 4
Preparation of libraries for cluster amplification and sequencing 4
Cluster amplification and sequencing 4
Data preprocessing 4

Characterization of candidate genes 6

rs117509001 (UBXN11) 6
rs2230288 (GBA) 6
rs2627037, rs922984, rs2291310, rs2291311, rs2291312 (TTN) 6
rs140485496 (IKBKB) 7
rs113574896 (MIR7705 / PABPC1 - Polyadenylate-binding protein 1) 7
chr10_105048270_AGAG_A (INA) 8
rs1865493 (KARS/TERF2IP) 8

Supplementary Figure S1 9

Supplementary Figure S2 10

Supplementary Figure S3 11

Supplementary Figure S4 12

Supplementary Figure S5 13

Supplementary Table S1 14

Supplementary Table S2 15

Supplementary Table S3 16

Supplementary Table S4 17

Supplementary Table S5 18

Supplementary Table S6 19

Supplementary Table S7 26

References 31

Acknowledgments

Exome sequencing of Finnish Parkinson's disease samples was supported by the Intramural Research Program of the National Institute on Aging, National Institutes of Health, Department of Health and Human Services [project Z01 AG000958-06]. The study was supported by grants from; Sigrid Juselius Foundation; The Finnish Parkinson Foundation.
The authors wish to acknowledge CSC – IT Center for Science, Finland, for computational resources. We also acknowledge the use of the gene set enrichment analysis, GSEA software, and Molecular Signature Database (MSigDB) (Subramanian, Tamayo, et al. (2005), PNAS 102, 15545-15550, http://www.broad.mit.edu/gsea/).

The NFBC1966 Study (Stampeed dataset) is conducted and supported by the National Heart, Lung, and Blood Institute (NHLBI) in collaboration with the Broad Institute, UCLA, University of Oulu, and the National Institute for Health and Welfare in Finland. This manuscript was not prepared in collaboration with investigators of the NFBC1966 Study and does not necessarily reflect the opinions or views of the NFBC1966 Study Investigators, Broad Institute, UCLA, University of Oulu, National Institute for Health and Welfare in Finland and the NHLBI.

FINRISK methods

FINRISK WES laboratory methods and data preparation

Whole exome libraries were constructed and sequenced on an Illumina HiSeq 4000 sequencer with the use of 151 bp paired-end reads. Output from Illumina software was processed by the Picard data-processing pipeline to yield BAM files containing well-calibrated, aligned reads. All sample information tracking was performed by automated LIMS messaging.

Library Construction

Library construction was performed as described by Fisher et al.[1] with some slight modifications. Initial genomic DNA input into shearing was reduced from 3μg to 50ng in 10μL of solution and enzymatically sheared. In addition, for adapter ligation, dual-indexed Illumina paired end adapters were replaced with palindromic forked adapters with unique 8 base index sequences embedded within the adapter and added to each end.

In-solution hybrid selection for exome enrichment

In-solution hybrid selection was performed using the Illumina Rapid Capture Exome enrichment kit with 38Mb target territory (29Mb baited). The targeted region included 98.3% of the intervals in the Refseq exome database. Dual-indexed libraries were pooled into groups of up to 96 samples prior to hybridization. The liquid handling was automated on a Hamilton Starlet. The enriched library pools were quantified via PicoGreen after elution from streptavadin beads and then normalized to a range compatible with sequencing template denature protocols.

Preparation of libraries for cluster amplification and sequencing

Following sample preparation, the libraries prepared using forked, indexed adapters were quantified using quantitative PCR (purchased from KAPA biosystems), normalized to 2 nM using the Hamilton Starlet Liquid Handling system, and pooled by equal volume using the Hamilton Starlet Liquid Handling system. Pools were then denatured using 0.1 N NaOH. Denatured samples were diluted into strip tubes using the the Hamilton Starlet Liquid Handling system.

Cluster amplification and sequencing

Cluster amplification of the templates was performed according to the manufacturer's protocol (Illumina) using the Illumina cBot. Flowcells were sequenced on HiSeq 4000 Sequencing-by-Synthesis Kits, then analyzed using RTA2.7.3

Data preprocessing

Raw sequencing data was processed from BCL to BAM. BAM files were processed to VCF files using best practices protocol of the Genome Analysis Toolkit (GATK, version 3.5 / Picard Toolkit, version

2). Briefly, all reads were used to reach exome deliverables. Samples were aligned to Human reference genome build version B37 using BWA MEM (version 0.0.7) with default settings. Samples with >= 10% contamination were excluded from call sets. Also, exome samples with less than 40% of targets at 20X coverage were excluded. HaplotypeCaller, GenotypeGVCF and VariantFiltration were applied.

Characterization of candidate genes

rs117509001 (UBXN11)

Variant rs117509001 is located in UBX domain-containing protein 11 (UBXN11). Variant is functionally synonymous and gnomAD allele frequency for European non-Finnish is 0.0074 and Finnish 0.0056. UBXN11 interacts with Rnd GTPases, activates Galpha(12) and may have a role in reorganization of actin cytoskeleton[2,3].

Interestingly, gene cluster with UBXN11 was found to be downregulated in frontal cortex area 8 in dementia with Lewy bodies (DLB) but not in Parkinson's disease (PD)[4] . In another study, one family with a rare neurological disorder, Aicardi syndrome, had UBXN11 with differential DNA methylation pattern[5] .

rs2230288 (GBA)

Variant rs2230288 is located in Glucosylceramidase (GBA) gene. This gene encodes enzyme in lysosome that is involved in breakdown of glycolipid glucosylceramide to ceramide and glucose. Mutations in this gene are associated to Gaucher disease, PD and DLB (see e.g. OMIM *606463). Variant rs2230288 is pathogenic for Gaucher's disease and has clinically controversial association to PD (Clinvar accessions VCV000199044 and VCV000004299). Variant is functionally missense mutation and gnomAD allele frequency for European non-Finnish is 0.01234 and Finnish 0.04325.

rs2627037, rs922984, rs2291310, rs2291311, rs2291312 (TTN)

Variants are located in Titin (TTN) gene. TTN is key component in striated muscles (OMIM *188840). Mutations in TTN gene are associated to cardiomyopathy, Salih Myopathy, Hereditary myopathy with early respiratory failure and muscular dystrophy. Variants rs2627037, rs922984, rs2291310, rs2291311 and rs2291312 are studied in relation to previously mentioned diseases and are classified as either benign or likely benign (Clinvar accessions VCV000047807.2, VCV000047736.2, VCV000046582.2, VCV000047692.2, VCV000047661.2). gnomAD allele frequencies for European non-Finnish and Finnish, respectively, are: rs2627037=0.07388/0.1097; rs922984=0.0709/0.1082; rs2291310=0.0696/0.1082; rs2291311=0.0696/0.1082; rs2291312=0.06894/0.1083. Variants locate in or in close proximity of Ig-like domains 19 and 20. These domains are behind titin's elasticity and it has been shown that also oxidation of the domains lead to stiffening of the protein[6].

Sequestosome 1 (SQSTM1) is found in several tauopathies and synucleinopathies, including PD[7]. Recent study found that SQSTM1 is phosphorylated by LRRK2 on Thr138 and that LRRK2 PD-associated mutations either increase or decrease the phosphorylation of SQSTM1[8]. SQSTM1 interacts with NBR1 and together they form a complex that target TK domain of TTN protein[9]. TTN mutation c.102271C>T p.Arg34091Trp at codon 279 (R279W) in TK domain, disrupts NBR1 interaction and leads to Hereditary myopathy with early respiratory failure.

rs140485496 (IKBKB)

Variant rs140485496 is located in gene Inhibitor of nuclear factor kappa-B kinase subunit beta (IKBKB). Gene is a serine kinase involved in activation of NF-kappa-B and belongs to I-kappa-B kinase (IKK) complex (OMIM * 603258). NF-kappa-B complex itself is involved in immune response, growth control and protection against apoptosis. Rs140485496 is a missense variant (NM_001556.2:c.1606C>T, p.Arg536Trp) and gnomAD allele frequency for European non-Finnish ancestry is 0.0004758 and for Finnish ancestry is 0.02279.

IKBKB is part of the E3 ubiquitin ligase TRAF6 signalling cascade[10]. TRAF6 is involved in TREM2 mediated neuroprotection by MAPK and NF-kappa-B signalling pathways in MPTP-induced neurotoxicity in PD mouse model[11]. Furthermore, TRAF6 together with SQSTM1 are required for polyubiquitination of p75 neurotrophin receptor which in turn rescues from amyloid beta-induced neuronal death in cell model[12]. The study suggested, that amyloid beta reduced interaction of IKBKB and p75 neurotrophin receptor and this resulted in downregulation of NF-kappa-B activity. Similarly, in a rotenone induced cell model of PD, siRNA mediated gene silencing of p75 protected against cell apoptosis[13].

It seems that IKBKB kinase activity is an essential part of NF-kappa-B regulation, and dysfunction of this system has negative effect for neuroprotection in MTPT, rotenone and amyloid beta –induced neurotoxicity in cell and animal models.

rs113574896 (MIR7705 / PABPC1 - Polyadenylate-binding protein 1)

Variant rs113574896 is a missense variant for Polyadenylate-binding protein 1 (PABPC1) and 2KB upstream variant for microRNA 7705 (MIR7705).

PABPC1 belongs to a PABP gene family that has role in mRNA poly(A) shortening and translation initiation (OMIM *604679). PABPC1 forms a complex with EIF4G1[14], a rare risk factor for PD[15], and EIF4G1 interacts with VPS35[16] and possibly with PINK1[17].

Variant rs113574896 in the *PABPC1* gene leads to p.L593V and is located in a MLLE (also known as PABC) domain. The domain binds regulatory proteins and translation factors that contain a conserved motif termed PAM2[18]. PABPC1 interacts with GIGYF2, a gene that has been associated with PD by linkage analysis, but subsequent replication studies have not been able to confirm this association[19-22], and TNRC6A has PAM2 motif, which suggests an interaction between TNRC6A and PABPC1. Overexpression of a yeast homolog of EIF4G1 or PABPC1 has been shown to suppress α-synuclein toxicity in a yeast model [23]. Furthermore, *eIF4E2*, a regulator of eIF4F complex[24] is located in the same locus 2q36-37 as GIGYF2 and they form a complex in mice[25]. Mutations in PABPC1 gene could disrupt the GIGYF2-TNRC6A-PABPC1-eIF4E2 interaction and result in dysregulation of eIF4F complex and/or microRNA-mediated gene silencing[26].

Interestingly, MIR7705 has an altered expression profile in retinal pigment cells under oxidative stress condition[27].

chr10_105048270_AGAG_A (INA)

Variation chr10_105048270_AGAG_A (rs760580716) is an inframe deletion, with allele frequency in gnomAD European non-Finnish ancestry is 0.0003201 and for Finnish ancestry is 0.00008323. Variation is located in alpha-internexin (INA) that is type IV neuronal intermediate filament (OMIM *605338).

INA is detected in neuronal intermediate filament inclusion disease (NIFID) inclusions and patients with NIFID may have parkinsonism[28] . INA has been found in inclusions in patients with Alzheimer's disease, dementia with Lewy body and motor neuron disease[29]. INA expression was found to be downregulated in patients with dementia with Lewy bodies[4]. Furthermore, posttranslational modification in INA was associated to Alzheimer's disease with cerebrovascular disease and altered polyubiquitinylation profile was observed in INA in patients with Alzheimer's disease and Down Syndrome[30,31] .

rs1865493 (KARS/TERF2IP)

Variant rs1865493 is located 5'UTR region of Telomeric repeat-binding factor 2-interacting protein 1 (TERF2IP) and within 2KB upstream of Lysine - tRNA ligase (KARS). Allele frequency in gnomAD European non-Finnish ancestry is 0.09379 and for Finnish ancestry is 0.1242. Interestingly, rs1865493 was associated to decreased telomere length[32].

TERF2IP is part of sheltering (telosome) complex[33,34] and is involved in telomere length and protection (OMIM *605061). TERF2IP protein levels are decreased when cells age and respond to oxidative stress[35]. Mice lacking both TERF2IP and telomerase compared to mice lacking only telomerase, show earlier onset of telomere-induced DNA damage and degenerative pathologies[36]. Recent meta-analysis did not find association between PD and telomere length[37] .

When cytoplasmic, TERF2IP also forms complex with IKK and induces pro-inflammatory cytokine production via NF-kappa-B signalling[38,39]. TERF2IP share a bidirectional promoter with lysyl-tRNA synthetase (KARS)[40].

KARS catalyzes attachment of aminoacid to tRNA-lys in the cytoplasm and mitochondria (OMIM * 601421). When secreted, enhances TNF-alpha production and triggers ERK, p38 MAPK and GNAI1 signalling pathways. Variants in KARS have been associated to neurological disorders such as Early-onset progressive microcephaly and Charcot-Marie-Tooth disease type 2[41,42]. Furthermore, mutations in KARS case also hypertrophic cardiomyopathy and mitochondrial respiratory chain defect[43]

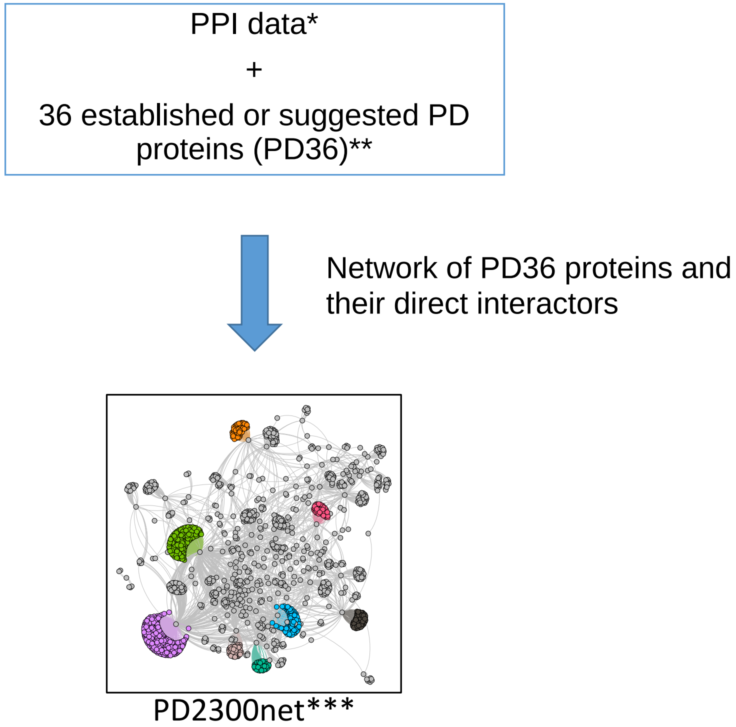

Supplementary Figure S1. Creation of PD2300net Protein-protein interaction network.

*Integrated interactions database (IID) with only experimentally detected human-specific interactions. In total the subset of the network contains 18627 UniProt protein ids and 280845 interactions.

** UniProt database (version 2018-02) was queried with the phrase *parkinson disease:disease AND organism:"Homo sapiens (Human) [9606]"*.

*** In total, the network (PD2300net) consists of 2305 UniProt protein identifiers representing 3413 interactions.

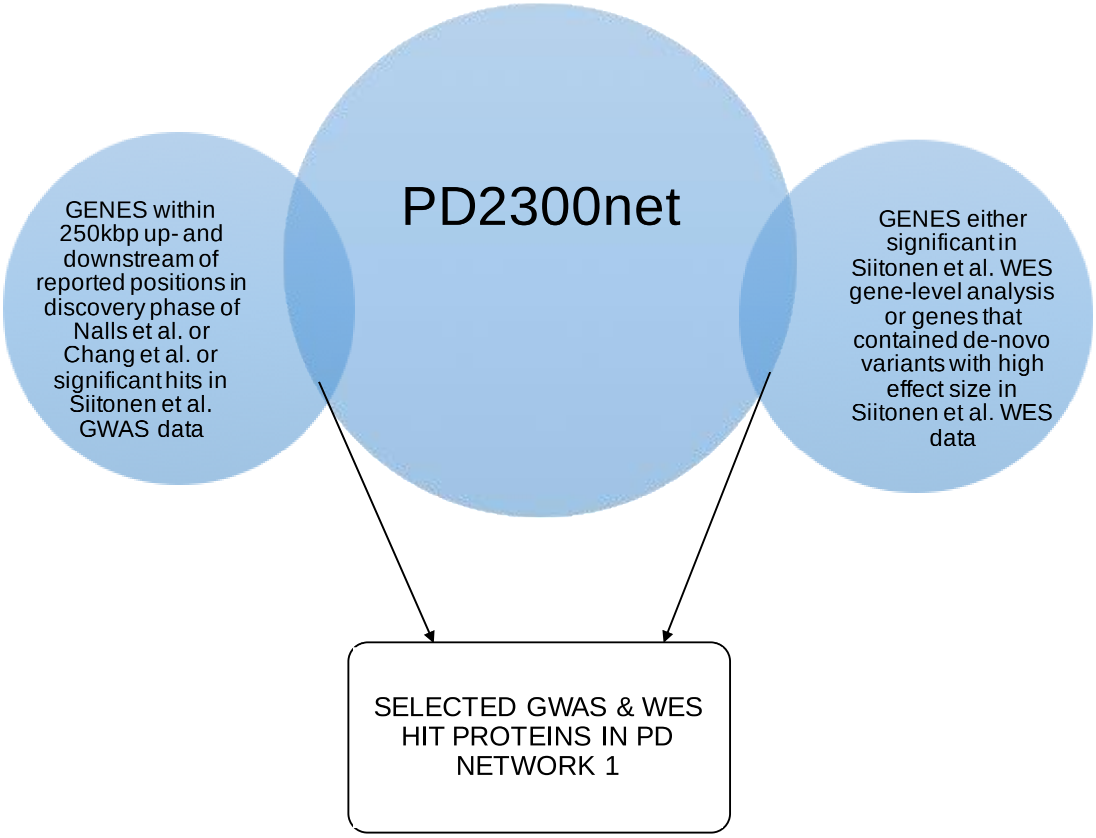

Supplementary Figure S2. Selecting proteins from GWAS and WES studies. Selected proteins are listed in Supplementary Table S1-S4.

1. Nalls MA, Pankratz N, Lill CM, et al. Large-scale meta-analysis of genome-wide association data identifies six new risk loci for parkinson's disease. *Nat Genet*. 2014;46(9):989-993.
2. Chang D, Nalls MA, Hallgrímsdóttir IB, et al. A meta-analysis of genome-wide association studies identifies 17 new parkinson's disease risk loci. *Nat Genet*. 2017;49(10):1511-1516. Accessed Nov 20, 2018. doi: 10.1038/ng.3955.
3. Siitonen A, Nalls MA, Hernández D. et al. Genetics of early-onset Parkinson's disease in Finland: exome sequencing and genome-wide association study. Neurobiol. Aging 53, 195.e-195.e10 (2017).

A)

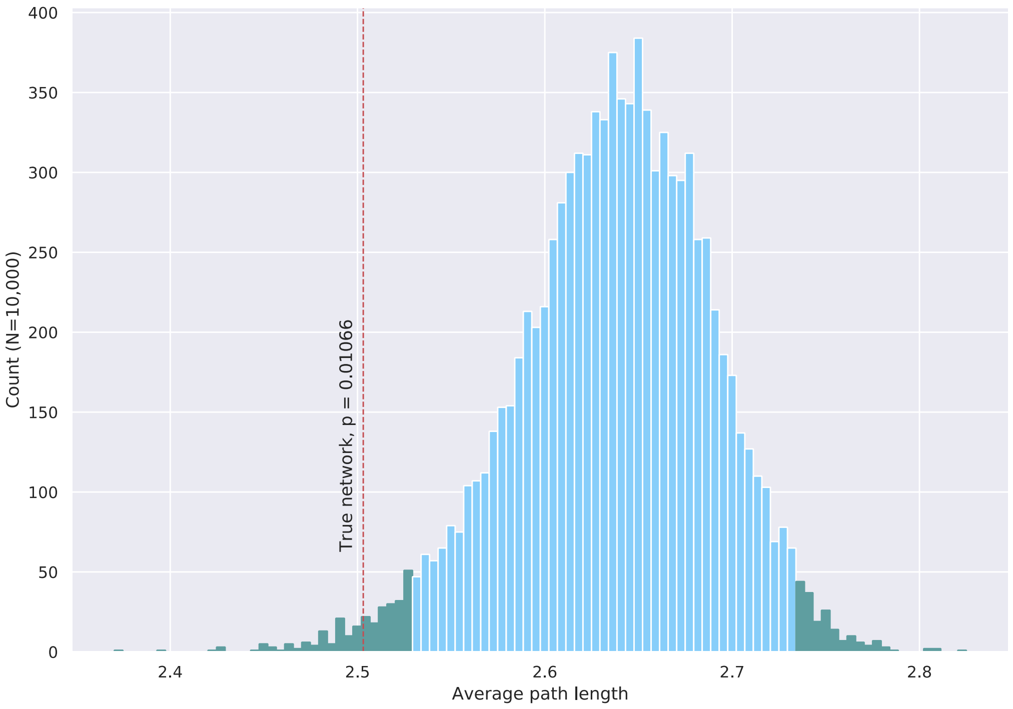

B)

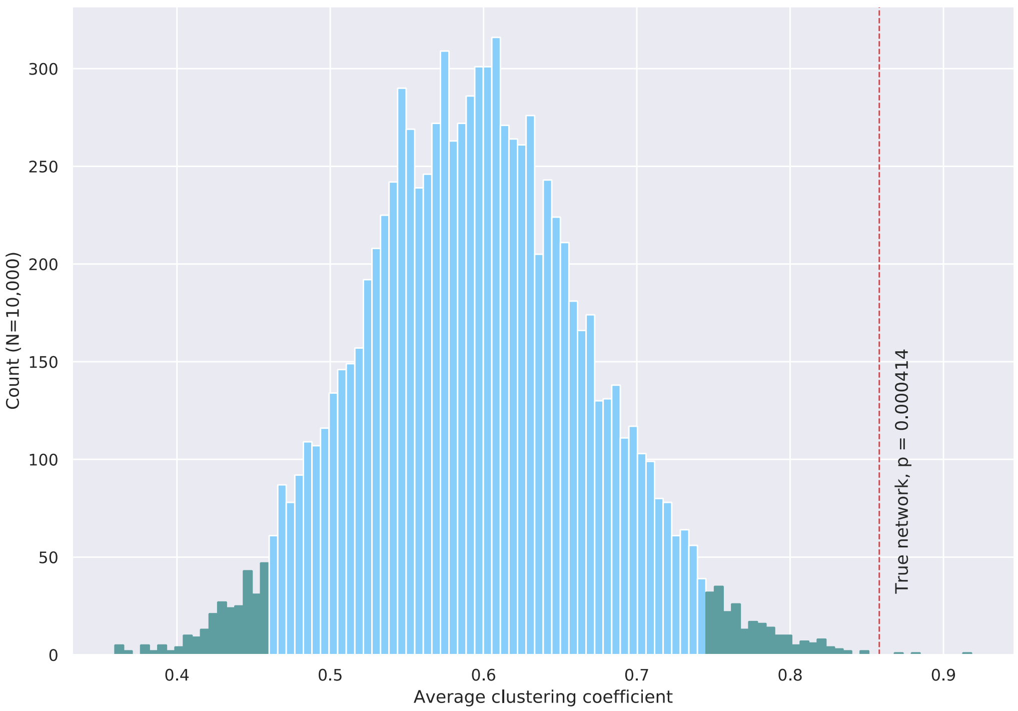

Supplementary Figure S3. Randomization tests for PD network 1. Count of randomized networks is 10,000. The 95% confidence intervals are colored in green. Value of the true network with one-sided p value is shown in red dash line. **A)** Average path length of true network ($p < 0.01066$) is shorter than what was expected of random networks with the same size and node degree distribution. **B)** Average clustering coefficient of true network ($p < 0.000414$) is higher than what was expected of random networks with the same size and node degree distribution.

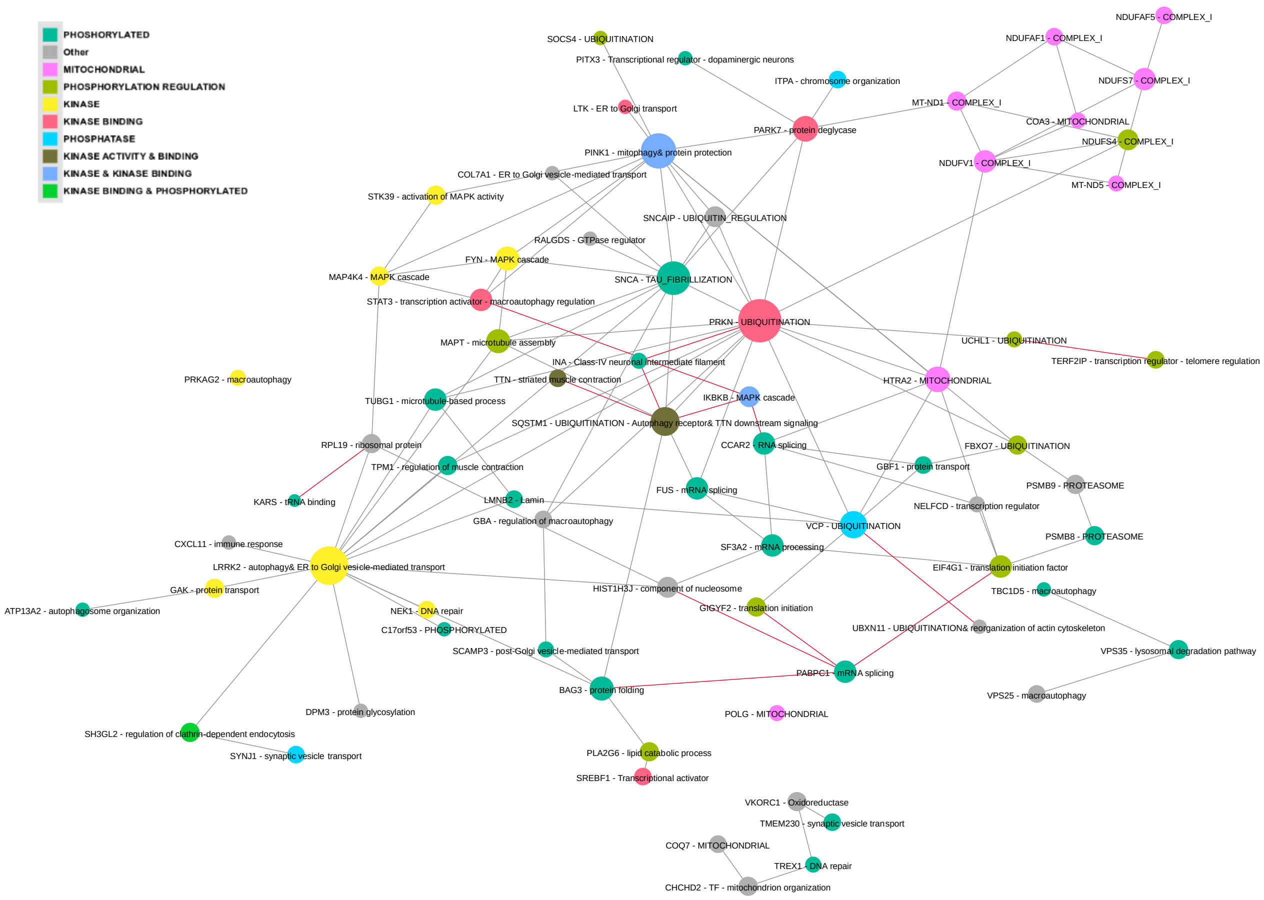

Supplementary Figure S4. Visualization of PD network 1 with information on phosphorylation and mitochondrial function. Edges of candidate proteins are highlighted in red.

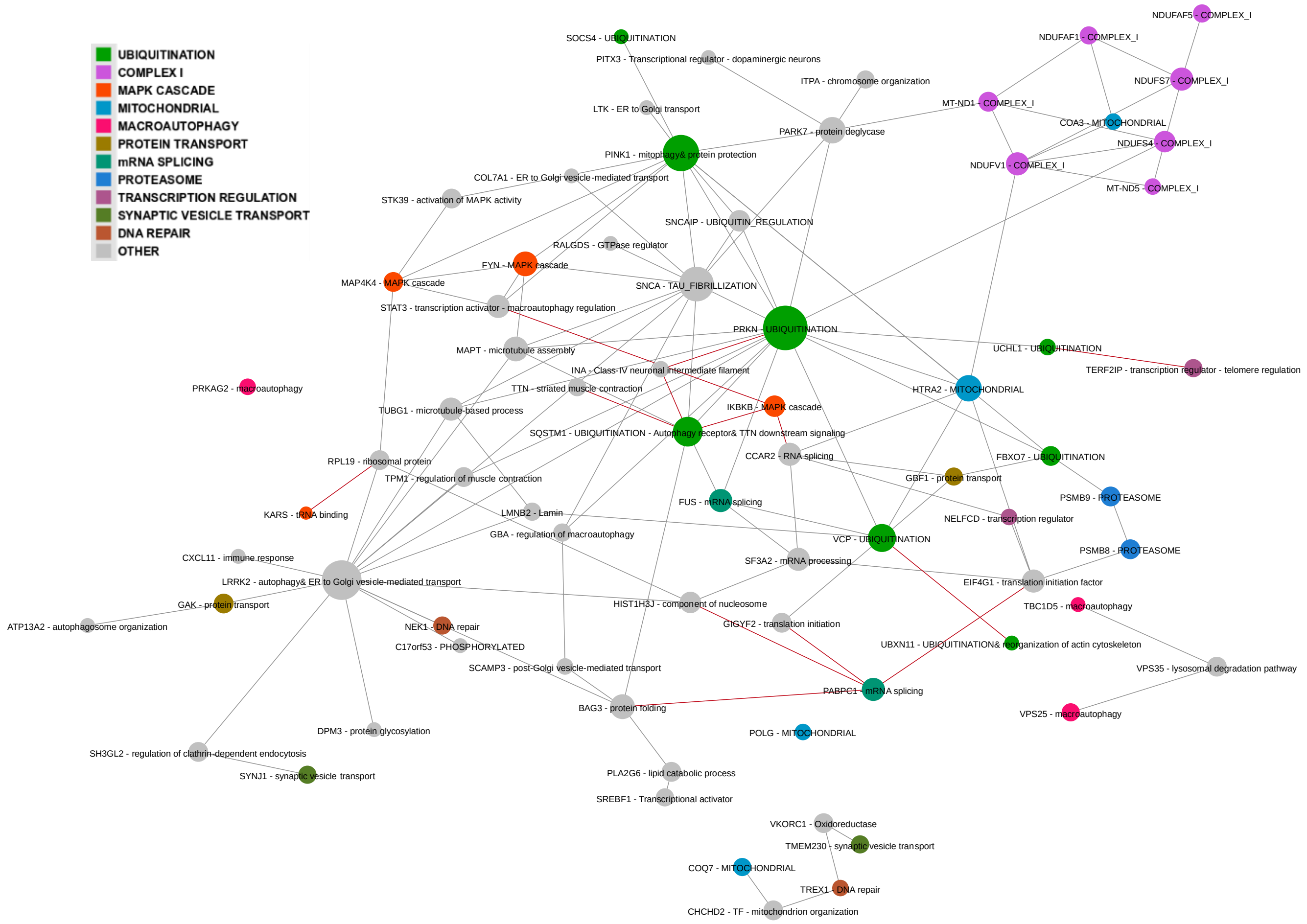

Supplementary Figure S5. Visualization of PD network 1 with information on Biological processes. Edges of candidate proteins are highlighted in red.

Supplementary Table S1. Reported GWAS chromosome positions in Nalls et al. and genes in PD2300net that are found within 250kbp up- and downstream of the positions.

Chromosome	Position	Genes
1	155135036	SCAMP3,DPM3,GBA
2	169110394	STK39
4	951947	GAK,TMEM175
4	77198986	CXCL11
4	90626111	SNCA
6	32666660	PSMB8,PSMB9
10	121536327	BAG3
12	40614434	LRRK2
14	55348869	SOCS4
15	61994134	VPS13C
16	31121793	FUS,VKORC1
17	17715101	SREBF1
17	43994648	MAPT
19	2363319	SF3A2,LMNB2
20	3168166	ITPA

Supplementary Table S2. Reported GWAS chromosome positions in Chang et al. and genes in PD2300net that are found within 250kbp up- and downstream of the positions.

**Chromosome**	**Position**	**Genes**
2	102413116	MAP4K4
3	18277488	TBC1D5
3	48748989	TREX1,COL7A1
4	170583157	NEK1
5	60273923	NDUFAF2
6	27681215	HIST1H3J
6	28054198	HIST1H3J
6	112242253	FYN
8	22525980	CCAR2
9	17579690	SH3GL2
10	104005410	GBF1,PITX3
15	41798614	LTK,NDUFAF1
15	63374825	TPM1
16	19279464	COQ7
17	17715101	SREBF1
17	40698158	VPS25,STAT3,TUBG1,COA3
17	42449789	C17orf53

Supplementary Table S3. Reported GWAS chromosome positions in Siitonen et al. and genes in PD2300net that are found within 250kbp up- and downstream of the positions.

Chromosome	Position	Genes
9	135955826	RALGDS

Supplementary Table S4. Reported WES genes in Siitonen et al. in PD2300net that were either significant in WES gene-level analyses or contained de-novo variants with high effect size.

UniProt ID	Gene	Selection criteria
P84098	RPL19	Significant in Collapsing burden test using EMMAX
Q8IXH7	NELFCD	Contained de-novo variant rs1043219

Supplementary Table S5. Proteins in PD network 1.

Uniprot ID	Gene Symbol	Uniprot ID	Gene Symbol
Q9NQ11	ATP13A2	Q8IXH7	NELFCD
O95817	BAG3	P11940	PABPC1
Q8N3J3	C17orf53	Q99497	PARK7
Q8N163	CCAR2	Q9BXM7	PINK1
Q9Y6H1	CHCHD2	O75364	PITX3
Q9Y2R0	COA3	O60733	PLA2G6
Q02388	COL7A1	P54098	POLG
Q99807	COQ7	Q9UGJ0	PRKAG2
O14625	CXCL11	O60260	PRKN
Q9P2X0	DPM3	P28062	PSMB8
Q04637	EIF4G1	P28065	PSMB9
Q9Y3I1	FBXO7	Q12967	RALGDS
P35637	FUS	P84098	RPL19
P06241	FYN	O14828	SCAMP3
O14976	GAK	Q15428	SF3A2
P04062	GBA	Q99962	SH3GL2
Q92538	GBF1	P37840	SNCA
Q6Y7W6	GIGYF2	Q9Y6H5	SNCAIP
P68431	HIST1H3J	Q8WXH5	SOCS4
O43464	HTRA2	Q13501	SQSTM1
O14920	IKBKB	P36956	SREBF1
Q16352	INA	P40763	STAT3
Q9BY32	ITPA	Q9UEW8	STK39
Q15046	KARS	O43426	SYNJ1
Q03252	LMNB2	Q92609	TBC1D5
Q5S007	LRRK2	Q9NYB0	TERF2IP
P29376	LTK	Q96A57	TMEM230
O95819	MAP4K4	P09493	TPM1
P10636	MAPT	Q9NSU2	TREX1
P03886	MT-ND1	Q8WZ42	TTN
P03915	MT-ND5	P23258	TUBG1
Q9Y375	NDUFAF1	Q5T124	UBXN11
Q5TEU4	NDUFAF5	P09936	UCHL1
O43181	NDUFS4	P55072	VCP
O75251	NDUFS7	Q9BQB6	VKORC1
P49821	NDUFV1	Q9BRG1	VPS25
Q96PY6	NEK1	Q96QK1	VPS35

Supplementary Table S6. GSEA gene sets in PD network 1.
[1]From String database Gene-set enrichment analysis. [2]From Broad Institutes GSEA analysis.

Gene Set Name[1]	*# Genes in Gene Set (K)*	*# Genes in Overlap (k)*	*k/K*	*p-value*	*FDR q-value*
GO:0006796 (phosphate-containing compound metabolic process)	2065	28	-	-	5.72e-08

**Gene Set Name[2]**	**# Genes in Gene Set (K)**	**# Genes in Overlap (k)**	**k/K**	**p-value**	**FDR q-value**
GO_MITOCHONDRION_ORGANIZATION	525	27	0,356944444	1.96E-31	1.96E-27
GO_REGULATION_OF_CATABOLIC_PROCESS	966	26	0,186805556	4.93E-23	2.46E-19
GO_PROCESS_UTILIZING_AUTOPHAGIC_MECHANISM	485	21	0,300694444	8.59E-23	2.86E-19
GO_REGULATION_OF_CELLULAR_CATABOLIC_PROCESS	812	24	0,205555556	3.64E-22	9.1E-19
GO_REGULATION_OF_AUTOPHAGY	319	17	0,370138889	4.84E-20	9.68E-17
GO_REGULATION_OF_MITOCHONDRION_ORGANIZATION	178	14	0,546527778	5.1E-19	8.49E-16
GO_MITOCHONDRION	1571	27	0,119444444	6.13E-19	8.75E-16
GO_PROTEIN_PHOSPHORYLATION	1967	29	0,102083333	1.29E-18	1.62E-15
GO_REGULATION_OF_PROTEIN_MODIFICATION_PROCESS	1843	28	0,105555556	2.83E-18	3.14E-15

GO_NEURON_PART	1715	27	0,109027778	5.58E-18	5.57E-15
GO_PROTEIN_CONTAINING_COMPLEX_ASSEMBLY	1911	28	0,102083333	7.22E-18	6.56E-15
GO_CELLULAR_MACROMOLECULE_CATABOLIC_PROCESS	1148	23	0,138888889	1.64E-17	1.37E-14
GO_REGULATION_OF_PHOSPHORUS_METABOLIC_PROCESS	1694	26	0,10625	5.04E-17	3.87E-14
GO_MACROMOLECULE_CATABOLIC_PROCESS	1375	24	0,121527778	6.25E-17	4.46E-14
GO_ORGANELLE_DISASSEMBLY	98	11	0,779166667	6.77E-17	4.51E-14
GO_NEURON_DEATH	347	15	0,3	2.08E-16	1.23E-13
GO_NEGATIVE_REGULATION_OF_NEURON_DEATH	209	13	0,431944444	2.21E-16	1.23E-13
GO_NEGATIVE_REGULATION_OF_PROTEIN_METABOLIC_PROCESS	1144	22	0,133333333	2.22E-16	1.23E-13
GO_POSITIVE_REGULATION_OF_PROTEIN_METABOLIC_PROCESS	1633	25	0,10625	2.51E-16	1.32E-13
GO_INTRACELLULAR_TRANSPORT	1825	26	0,098611111	3.00E-16	1.5E-13
GO_REGULATION_OF_RESPONSE_TO_STRESS	1497	24	0,111111111	4.17E-16	1.98E-13
GO_POSITIVE_REGULATION_OF_CELLULAR_COMPONENT_ORGANIZATION	1191	22	0,128472222	5.1E-16	2.25E-13
GO_MACROAUTOPHAGY	291	14	0,334027778	5.17E-16	2.25E-13
GO_CATALYTIC_COMPLEX	1368	23	0,116666667	7.18E-16	2.99E-13
GO_MITOCHONDRIAL_MEMBRANE_PART	231	13	0,390972222	8.16E-16	3.26E-13

GO_MITOCHONDRIAL_ELECTRON_TRANSPORT_NADH_TO_UBIQUINONE	53	9	1,179166667	9.93E-16	3.82E-13
GO_REGULATION_OF_PROTEOLYSIS	720	18	0,173611111	2.12E-15	7.84E-13
GO_CELLULAR_PROTEIN_CATABOLIC_PROCESS	760	18	0,164583333	5.36E-15	1.91E-12
GO_ENVELOPE	1180	21	0,123611111	5.59E-15	1.93E-12
GO_PROTEIN_CATABOLIC_PROCESS	911	19	0,145138889	8.24E-15	2.75E-12
GO_CELL_BODY	559	16	0,198611111	1.17E-14	3.76E-12
GO_REGULATION_OF_AUTOPHAGY_OF_MITOCHONDRION	41	8	1,354861111	1.26E-14	3.87E-12
GO_PEPTIDYL_AMINO_ACID_MODIFICATION	1231	21	0,11875	1.28E-14	3.87E-12
GO_RESPONSE_TO_OXYGEN_CONTAINING_COMPOUND	1616	23	0,098611111	2.45E-14	7.21E-12
GO_AUTOPHAGY_OF_MITOCHONDRION	75	9	0,833333333	2.72E-14	7.77E-12
GO_NEURON_PROJECTION	1304	21	0,111805556	3.91E-14	1.09E-11
GO_NEGATIVE_REGULATION_OF_PROTEIN_MODIFICATION_PROCESS	610	16	0,181944444	4.46E-14	1.2E-11
GO_REGULATION_OF_KINASE_ACTIVITY	864	18	0,144444444	4.77E-14	1.25E-11
GO_NEGATIVE_REGULATION_OF_CELL_DEATH	1019	19	0,129166667	6.07E-14	1.56E-11
GO_REGULATION_OF_CELLULAR_LOCALIZATION	886	18	0,140972222	7.29E-14	1.82E-11
GO_MITOCHONDRIAL_PART	1033	19	0,127777778	7.74E-14	1.89E-11

GO_REGULATION_OF_CELL_DEATH	1723	23	0,092361 111	9.38E-14	2.23E-11
GO_MITOCHONDRIAL_RESPIRATORY_CHAIN_COMPLEX_I	52	8	1,068055 556	9.72E-14	2.26E-11
GO_ORGANELLE_INNER_MEMBRANE	530	15	0,196527 778	1.01E-13	2.31E-11
GO_CELLULAR_RESPIRATION	191	11	0,4	1.21E-13	2.68E-11
GO_PROTEOLYSIS	1762	23	0,090972 222	1.49E-13	3.25E-11
GO_RESPONSE_TO_OXIDATIVE_STRESS	447	14	0,217361 111	1.87E-13	3.97E-11
GO_OXIDATIVE_PHOSPHORYLATION	140	10	0,495833 333	1.93E-13	4.01E-11
GO_NEGATIVE_REGULATION_OF_PHOSPHORUS_METABOLIC_PROCESS	557	15	0,186805 556	2.07E-13	4.21E-11
GO_NEGATIVE_REGULATION_OF_PHOSPHORYLATION	451	14	0,215277 778	2.11E-13	4.21E-11
GO_REGULATION_OF_PROTEIN_STABILITY	276	12	0,302083 333	2.45E-13	4.81E-11
GO_ORGANONITROGEN_COMPOUND_CATABOLIC_PROCESS	1268	20	0,109722 222	2.62E-13	5.03E-11
GO_ATP_SYNTHESIS_COUPLED_ELECTRON_TRANSPORT	96	9	0,651388 889	2.72E-13	5.13E-11
GO_REGULATION_OF_TRANSFERASE_ACTIVITY	963	18	0,129861 111	2.97E-13	5.5E-11
GO_ENERGY_DERIVATION_BY_OXIDATION_OF_ORGANIC_COMPOUNDS	281	12	0,296527 778	3.03E-13	5.51E-11
GO_CELLULAR_PROTEIN_CONTAINING_COMPLEX_ASSEMBLY	1130	19	0,116666 667	3.77E-13	6.72E-11
GO_BEHAVIOR	594	15	0,175694 444	5.22E-13	9.15E-11

GO_CELLULAR_RESPONSE_TO_OXIDATIVE_STRESS	299	12	0,278472222	6.31E-13	1.09E-10
GO_REGULATION_OF_CELLULAR_RESPONSE_TO_STRESS	730	16	0,152083333	6.84E-13	1.16E-10
GO_SYNAPSE	1171	19	0,1125	7.04E-13	1.16E-10
GO_NEGATIVE_REGULATION_OF_CATABOLIC_PROCESS	302	12	0,275694444	7.1E-13	1.16E-10
GO_IDENTICAL_PROTEIN_BINDING	1711	22	0,089583333	7.76E-13	1.25E-10
GO_MITOCHONDRIAL_ENVELOPE	739	16	0,150694444	8.24E-13	1.31E-10
GO_MODIFICATION_DEPENDENT_MACROMOLECULE_CATABOLIC_PROCESS	626	15	0,166666667	1.1E-12	1.72E-10
GO_REGULATION_OF_MACROAUTOPHAGY	168	10	0,413194444	1.21E-12	1.86E-10
GO_RESPIRATORY_ELECTRON_TRANSPORT_CHAIN	115	9	0,54375	1.43E-12	2.17E-10
GO_POSITIVE_REGULATION_OF_PROTEIN_MODIFICATION_PROCESS	1221	19	0,108333333	1.46E-12	2.17E-10
GO_NEGATIVE_REGULATION_OF_SIGNALING	1394	20	0,099305556	1.48E-12	2.17E-10
GO_POSITIVE_REGULATION_OF_CATABOLIC_PROCESS	418	13	0,215972222	1.61E-12	2.33E-10
GO_PROTEIN_LOCALIZATION_TO_ORGANELLE	919	17	0,128472222	1.77E-12	2.52E-10
GO_NEGATIVE_REGULATION_OF_CELLULAR_CATABOLIC_PROCESS	247	11	0,309027778	2.01E-12	2.83E-10
GO_POSITIVE_REGULATION_OF_PHOSPHORUS_METABOLIC_PROCESS	1084	18	0,115277778	2.14E-12	2.98E-10
GO_CELLULAR_COMPONENT_DISASSEMBLY	538	14	0,180555556	2.26E-12	3.08E-10

GO_SYNAPSE_PART	934	17	0,126388889	2.28E-12	3.08E-10
GO_REGULATION_OF_ORGANELLE_ORGANIZATION	1259	19	0,104861111	2.49E-12	3.32E-10
GO_REGULATION_OF_PEPTIDASE_ACTIVITY	443	13	0,203472222	3.33E-12	4.38E-10
GO_INCLUSION_BODY	81	8	0,686111111	3.97E-12	5.16E-10
GO_CELLULAR_RESPONSE_TO_OXYGEN_CONTAINING_COMPOUND	1126	18	0,111111111	4.02E-12	5.16E-10
GO_POSITIVE_REGULATION_OF_PROTEOLYSIS	353	12	0,236111111	4.41E-12	5.58E-10
GO_NEGATIVE_REGULATION_OF_CELLULAR_COMPONENT_ORGANIZATION	700	15	0,148611111	5.39E-12	6.73E-10
GO_REGULATION_OF_PROTEIN_LOCALIZATION	990	17	0,119444444	5.71E-12	7.05E-10
GO_CELLULAR_MACROMOLECULE_LOCALIZATION	1897	22	0,080555556	5.91E-12	7.2E-10
GO_RESPIRATORY_CHAIN_COMPLEX	86	8	0,645833333	6.5E-12	7.83E-10
GO_REGULATION_OF_ESTABLISHMENT_OF_PROTEIN_LOCALIZATION	730	15	0,142361111	9.73E-12	1.16E-9
GO_POSITIVE_REGULATION_OF_MOLECULAR_FUNCTION	1756	21	0,083333333	1.13E-11	1.33E-9
GO_GENERATION_OF_PRECURSOR_METABOLITES_AND_ENERGY	502	13	0,179861111	1.58E-11	1.84E-9
GO_MITOCHONDRIAL_RESPIRATORY_CHAIN_COMPLEX_ASSEMBLY	96	8	0,578472222	1.6E-11	1.84E-9
GO_NEGATIVE_REGULATION_OF_RESPONSE_TO_OXIDATIVE_STRESS	56	7	0,868055556	1.69E-11	1.92E-9
GO_REGULATION_OF_CELLULAR_COMPONENT_BIOGENESIS	917	16	0,120833333	2.07E-11	2.33E-9

GO_POSITIVE_REGULATION_OF_SIGNALING	1828	21	0,079861111	2.4E-11	2.66E-9
GO_RESPIRASOME	101	8	0,55	2.42E-11	2.66E-9
GO_REGULATION_OF_CELLULAR_PROTEIN_LOCALIZATION	522	13	0,172916667	2.56E-11	2.79E-9
GO_REGULATION_OF_TRANSPORT	1842	21	0,079166667	2.76E-11	2.97E-9
GO_CELL_CELL_SIGNALING	1644	20	0,084722222	2.89E-11	3.07E-9
GO_REGULATION_OF_HYDROLASE_ACTIVITY	1277	18	0,097916667	3.17E-11	3.34E-9
GO_NEGATIVE_REGULATION_OF_RESPONSE_TO_STIMULUS	1655	20	0,084027778	3.25E-11	3.38E-9
GO_POSITIVE_REGULATION_OF_TRANSPORT	955	16	0,116666667	3.77E-11	3.89E-9
GO_NADH_DEHYDROGENASE_COMPLEX_ASSEMBLY	64	7	0,759722222	4.47E-11	4.56E-9
GO_SOMATODENDRITIC_COMPARTMENT	820	15	0,127083333	4.95E-11	5.00E-09
GO_REGULATION_OF_CELLULAR_PROTEIN_CATABOLIC_PROCESS	248	10	0,279861111	5.74E-11	5.74E-9

Supplementary Table S7. Functional characterisation of the candidate genes.

Variant	Gene	gnomAD non-Finnish European	gnomAD Finnish	Gene's Link to diseases, especially neurologic	Type of association	References
**rs117509001**	UBXN11	0.0074	0.0056	dementia with Lewy bodies (DLB)	Gene expression	(Santpere, Garcia-Esparcia et al. 2018)
				Aicardi syndrome	Methylation	(Piras, Mills et al. 2017)
**rs2230288**	GBA	0.01234	0.04325	Gaucher's disease, PD	Genetic	(Huang, Deng et al. 2018, Jmoudiak, Futerman 2005)
**rs2627037, rs922984, rs2291310, rs2291311, rs2291312**	TTN	0.07388, 0.0709, 0.0696, 0.0696, 0.06894	0.1097, 0.1082, 0.1082, 0.1082, 0.1083	Hereditary myopathy with early respiratory failure	Genetic, cell model	(Lange, Xiang et al. 2005)
				Synucleinopathies indirectly via SQSTM1/NBR1 interaction?	Protein location in inclusions in cell model. Co-immunoprecipitation	(Lange, Xiang et al. 2005, Kuusisto, Salminen et al. 2001)
**rs140485496**	IKBKB	0.0004758	0.02279	Indirectly to PD via TRAF6/TREM2/SQSTM1/p75 signalling cascade	MPTP-mouse model & Rotenone cell model	(Yoboua, Martel et al. 2010, Ren, Guo et al. 2018, Chen, Hou et al. 2018)
**rs113574896**	PABPC1	0.0003735	0.0001212	Indirectly to synucleinopathies via GIGYF2-TNRC6A-PABPC1-eIF4E2 interaction	Yeast model. Protein structure. Protein fragments complementation assay. Mass spectrometry	(Kozlov, Gehring 2010, Schopp, Amaya Ramirez et al. 2017, Tarun, Sachs 1996, Khurana, Peng et al. 2017, Morita,

Variant	Gene	gnomAD non-Finnish European	gnomAD Finnish	Gene's Link to diseases, especially neurologic	Type of association	References
						Ler et al. 2012, Nishi, Nishi et al. 2013)
**chr10_105048270_AGAG_A**	INA	0.0003201	0.00008323	neuronal intermediate filament inclusion disease with parkinsonism	Genetic	(Cairns, Grossman et al. 2004)
				inclusions in patients with Alzheimer's disease, dementia with Lewy body and motor neuron disease	Protein location in inclusions in post-mortem brains.	(Cairns, Uryu et al. 2004)
				downregulated in patients with dementia with Lewy bodies	RT-qPCR	(Santpere, Garcia-Esparcia et al. 2018)
				Down syndrome with Alzheimer's disease	Altered polyubiquitinylation profile in postmortem brains	(Tramutola, Di Domenico et al. 2017)
**rs1865493**	TERF2IP	0.09379	0.1242	rs1865493 was associated to decreased telomere length	Genetic with qPCR	(You, Chen et al. 2012)
				earlier onset of telomere-induced DNA damage and degenerative pathologies	Mouse model with histopathological and telomere quantitative FISH analyses	(Martinez, Gomez-Lopez et al. 2016)
**rs1865493**	KARS	0.09379	0.1242	Early-onset progressive microcephaly	Genetic	(McMillan, Humphreys et al. 2015)
				Charcot-Marie-Tooth disease type 2		

Variant	Gene	gnomAD non-Finnish European	gnomAD Finnish	Gene’s Link to diseases, especially neurologic	Type of association	References
					Neuropathy in charcot-marie-tooth 2D mouse model	(Seburn, Nangle et al. 2006)
				hypertrophic cardiomyopathy and mitochondrial respiratory chain defect	Genetic with muscle biopsy	(Verrigni, Diodato et al. 2017)

References in functional characterisation of the candidate genes

CAIRNS, N.J., GROSSMAN, M., ARNOLD, S.E., BURN, D.J., JAROS, E., PERRY, R.H., DUYCKAERTS, C., STANKOFF, B., PILLON, B., SKULLERUD, K., CRUZ-SANCHEZ, F.F., BIGIO, E.H., MACKENZIE, I.R., GEARING, M., JUNCOS, J.L., GLASS, J.D., YOKOO, H., NAKAZATO, Y., MOSAHEB, S., THORPE, J.R., URYU, K., LEE, V.M. and TROJANOWSKI, J.Q., 2004. Clinical and neuropathologic variation in neuronal intermediate filament inclusion disease. *Neurology,* **63**(8), pp. 1376-1384.

CAIRNS, N.J., URYU, K., BIGIO, E.H., MACKENZIE, I.R., GEARING, M., DUYCKAERTS, C., YOKOO, H., NAKAZATO, Y., JAROS, E., PERRY, R.H., ARNOLD, S.E., LEE, V.M. and TROJANOWSKI, J.Q., 2004. alpha-Internexin aggregates are abundant in neuronal intermediate filament inclusion disease (NIFID) but rare in other neurodegenerative diseases. *Acta Neuropathologica,* **108**(3), pp. 213-223.

CHEN, Y., HOU, Y., YANG, J., DU, R., CHEN, C., CHEN, F., WANG, H., GE, R. and CHEN, J., 2018. P75 Involved in the Ubiquitination of alpha-synuclein in Rotenone-based Parkinson's Disease Models. *Neuroscience,* **388**, pp. 367-373.

HUANG, Y., DENG, L., ZHONG, Y. and YI, M., 2018. The Association between E326K of GBA and the Risk of Parkinson's Disease. *Parkinson's disease,* **2018**, pp. 1048084.

JMOUDIAK, M. and FUTERMAN, A.H., 2005. Gaucher disease: pathological mechanisms and modern management. *British journal of haematology,* **129**(2), pp. 178-188.

KHURANA, V., PENG, J., CHUNG, C.Y., AULUCK, P.K., FANNING, S., TARDIFF, D.F., BARTELS, T., KOEVA, M., EICHHORN, S.W., BENYAMINI, H., LOU, Y., NUTTER-UPHAM, A., BARU, V., FREYZON, Y., TUNCBAG, N., COSTANZO, M., SAN LUIS, B.J., SCHONDORF, D.C., BARRASA, M.I., EHSANI, S., SANJANA, N., ZHONG, Q., GASSER, T., BARTEL, D.P., VIDAL, M., DELEIDI, M., BOONE, C., FRAENKEL, E., BERGER, B. and LINDQUIST, S., 2017. Genome-Scale Networks Link Neurodegenerative Disease Genes to alpha-Synuclein through Specific Molecular Pathways. *Cell systems,* **4**(2), pp. 170.e14.

KOZLOV, G. and GEHRING, K., 2010. Molecular basis of eRF3 recognition by the MLLE domain of poly(A)-binding protein. *PloS one,* **5**(4), pp. e10169.

KUUSISTO, E., SALMINEN, A. and ALAFUZOFF, I., 2001. Ubiquitin-binding protein p62 is present in neuronal and glial inclusions in human tauopathies and synucleinopathies. *Neuroreport,* **12**(10), pp. 2085-2090.

LANGE, S., XIANG, F., YAKOVENKO, A., VIHOLA, A., HACKMAN, P., ROSTKOVA, E., KRISTENSEN, J., BRANDMEIER, B., FRANZEN, G., HEDBERG, B., GUNNARSSON, L.G., HUGHES, S.M., MARCHAND, S., SEJERSEN, T., RICHARD, I., EDSTROM, L., EHLER, E., UDD, B. and GAUTEL, M., 2005. The kinase domain of titin controls muscle gene expression and protein turnover. *Science (New York, N.Y.),* **308**(5728), pp. 1599-1603.

MARTINEZ, P., GOMEZ-LOPEZ, G., PISANO, D.G., FLORES, J.M. and BLASCO, M.A., 2016. A genetic interaction between RAP1 and telomerase reveals an unanticipated role for RAP1 in telomere maintenance. *Aging cell,* **15**(6), pp. 1113-1125.

MCMILLAN, H.J., HUMPHREYS, P., SMITH, A., SCHWARTZENTRUBER, J., CHAKRABORTY, P., BULMAN, D.E., BEAULIEU, C.L., FORGE CANADA CONSORTIUM, MAJEWSKI, J., BOYCOTT, K.M. and GERAGHTY, M.T., 2015. Congenital Visual Impairment and Progressive Microcephaly Due to Lysyl-Transfer Ribonucleic Acid (RNA) Synthetase (KARS) Mutations: The Expanding Phenotype of Aminoacyl-Transfer RNA Synthetase Mutations in Human Disease. *Journal of child neurology,* **30**(8), pp. 1037-1043.

MORITA, M., LER, L.W., FABIAN, M.R., SIDDIQUI, N., MULLIN, M., HENDERSON, V.C., ALAIN, T., FONSECA, B.D., KARASHCHUK, G., BENNETT, C.F., KABUTA, T., HIGASHI, S., LARSSON, O., TOPISIROVIC, I., SMITH, R.J., GINGRAS, A.C. and SONENBERG, N., 2012. A novel 4EHP-GIGYF2 translational repressor complex is essential for mammalian development. *Molecular and cellular biology,* **32**(17), pp. 3585-3593.

NISHI, K., NISHI, A., NAGASAWA, T. and UI-TEI, K., 2013. Human TNRC6A is an Argonaute-navigator protein for microRNA-mediated gene silencing in the nucleus. *RNA (New York, N.Y.),* **19**(1), pp. 17-35.

PIRAS, I.S., MILLS, G., LLACI, L., NAYMIK, M., RAMSEY, K., BELNAP, N., BALAK, C.D., JEPSEN, W.M., SZELINGER, S., SINIARD, A.L., LEWIS, C.R., LAFLEUR, M., RICHHOLT, R.F., DE BOTH, M.D., AVELA, K., RANGASAMY, S., CRAIG, D.W., NARAYANAN, V., JARVELA, I., HUENTELMAN, M.J. and SCHRAUWEN, I., 2017. Exploring genome-wide DNA methylation patterns in Aicardi syndrome. *Epigenomics,* **9**(11), pp. 1373-1386.

REN, M., GUO, Y., WEI, X., YAN, S., QIN, Y., ZHANG, X., JIANG, F. and LOU, H., 2018. TREM2 overexpression attenuates neuroinflammation and protects dopaminergic neurons in experimental models of Parkinson's disease. *Experimental neurology,* **302**, pp. 205-213.

SANTPERE, G., GARCIA-ESPARCIA, P., ANDRES-BENITO, P., LORENTE-GALDOS, B., NAVARRO, A. and FERRER, I., 2018. Transcriptional network analysis in frontal cortex in Lewy body diseases with focus on dementia with Lewy bodies. *Brain pathology (Zurich, Switzerland),* **28**(3), pp. 315-333.

SCHOPP, I.M., AMAYA RAMIREZ, C.C., DEBELJAK, J., KREIBICH, E., SKRIBBE, M., WILD, K. and BETHUNE, J., 2017. Split-BioID a conditional proteomics approach to monitor the composition of spatiotemporally defined protein complexes. *Nature communications,* **8**, pp. 15690.

SEBURN, K.L., NANGLE, L.A., COX, G.A., SCHIMMEL, P. and BURGESS, R.W., 2006. An active dominant mutation of glycyl-tRNA synthetase causes neuropathy in a Charcot-Marie-Tooth 2D mouse model. *Neuron,* **51**(6), pp. 715-726.

TARUN, S.Z.,Jr and SACHS, A.B., 1996. Association of the yeast poly(A) tail binding protein with translation initiation factor eIF-4G. *The EMBO journal,* **15**(24), pp. 7168-7177.

TRAMUTOLA, A., DI DOMENICO, F., BARONE, E., ARENA, A., GIORGI, A., DI FRANCESCO, L., SCHININA, M.E., COCCIA, R., HEAD, E., BUTTERFIELD, D.A. and PERLUIGI, M., 2017. Polyubiquitinylation Profile in Down Syndrome Brain Before and After the Development of Alzheimer Neuropathology. *Antioxidants & redox signaling,* **26**(7), pp. 280-298.

VERRIGNI, D., DIODATO, D., DI NOTTIA, M., TORRACO, A., BELLACCHIO, E., RIZZA, T., TOZZI, G., VERARDO, M., PIEMONTE, F., TASCA, G., D'AMICO, A., BERTINI, E. and CARROZZO, R., 2017. Novel mutations in KARS cause hypertrophic cardiomyopathy and combined mitochondrial respiratory chain defect. *Clinical genetics,* **91**(6), pp. 918-923.

YOBOUA, F., MARTEL, A., DUVAL, A., MUKAWERA, E. and GRANDVAUX, N., 2010. Respiratory syncytial virus-mediated NF-kappa B p65 phosphorylation at serine 536 is dependent on RIG-I, TRAF6, and IKK beta. *Journal of virology,* **84**(14), pp. 7267-7277.

YOU, N.C., CHEN, B.H., SONG, Y., LU, X., CHEN, Y., MANSON, J.E., KANG, M., HOWARD, B.V., MARGOLIS, K.L., CURB, J.D., PHILLIPS, L.S., STEFANICK, M.L., TINKER, L.F. and LIU, S., 2012. A prospective study of leukocyte telomere length and risk of type 2 diabetes in postmenopausal women. *Diabetes,* **61**(11), pp. 2998-3004.

References

1. Fisher S, Barry A, Abreu J, et al. A scalable, fully automated process for construction of sequence-ready human exome targeted capture libraries. *Genome Biol*. 2011;12(1):R1. Accessed Nov 21, 2018. doi: 10.1186/gb-2011-12-1-r1.

2. Tateiwa K, Katoh H, Negishi M. Socius, a novel binding partner of Galpha12/13, promotes the Galpha12-induced RhoA activation. *Biochem Biophys Res Commun*. 2005;337(2):615-620. doi: S0006-291X(05)02125-X [pii].

3. Katoh H, Harada A, Mori K, Negishi M. Socius is a novel rnd GTPase-interacting protein involved in disassembly of actin stress fibers. *Mol Cell Biol*. 2002;22(9):2952-2964.

4. Santpere G, Garcia-Esparcia P, Andres-Benito P, Lorente-Galdos B, Navarro A, Ferrer I. Transcriptional network analysis in frontal cortex in lewy body diseases with focus on dementia with lewy bodies. *Brain Pathol*. 2018;28(3):315-333. doi: 10.1111/bpa.12511 [doi].

5. Piras IS, Mills G, Llaci L, et al. Exploring genome-wide DNA methylation patterns in aicardi syndrome. *Epigenomics*. 2017;9(11):1373-1386. doi: 10.2217/epi-2017-0060 [doi].

6. Giganti D, Yan K, Badilla CL, Fernandez JM, Alegre-Cebollada J. Disulfide isomerization reactions in titin immunoglobulin domains enable a mode of protein elasticity. *Nat Commun*. 2018;9(1):7. doi: 10.1038/s41467-017-02528-7 [doi].

7. Kuusisto E, Salminen A, Alafuzoff I. Ubiquitin-binding protein p62 is present in neuronal and glial inclusions in human tauopathies and synucleinopathies. *Neuroreport*. 2001;12(10):2085-2090.

8. Kalogeropulou AF, Zhao J, Bolliger MF, et al. P62/SQSTM1 is a novel leucine-rich repeat kinase 2 (LRRK2) substrate that enhances neuronal toxicity. *Biochem J*. 2018;475(7):1271-1293. doi: 10.1042/BCJ20170699 [doi].

9. Lange S, Xiang F, Yakovenko A, et al. The kinase domain of titin controls muscle gene expression and protein turnover. *Science*. 2005;308(5728):1599-1603. doi: 1110463 [pii].

10. Yoboua F, Martel A, Duval A, Mukawera E, Grandvaux N. Respiratory syncytial virus-mediated NF-kappa B p65 phosphorylation at serine 536 is dependent on RIG-I, TRAF6, and IKK beta. *J Virol*. 2010;84(14):7267-7277. doi: 10.1128/JVI.00142-10 [doi].

11. Ren M, Guo Y, Wei X, et al. TREM2 overexpression attenuates neuroinflammation and protects dopaminergic neurons in experimental models of parkinson's disease. *Exp Neurol*. 2018;302:205-213. doi: S0014-4886(18)30023-2 [pii].

12. Geetha T, Zheng C, McGregor WC, et al. TRAF6 and p62 inhibit amyloid beta-induced neuronal death through p75 neurotrophin receptor. *Neurochem Int*. 2012;61(8):1289-1293. doi: 10.1016/j.neuint.2012.09.005 [doi].

13. Chen Y, Hou Y, Yang J, et al. P75 involved in the ubiquitination of alpha-synuclein in rotenone-based parkinson's disease models. *Neuroscience*. 2018;388:367-373. doi: S0306-4522(18)30530-X [pii].

14. Tarun SZ,Jr, Sachs AB. Association of the yeast poly(A) tail binding protein with translation initiation factor eIF-4G. *EMBO J*. 1996;15(24):7168-7177.

15. Nichols N, Bras JM, Hernandez DG, et al. EIF4G1 mutations do not cause parkinson's disease. *Neurobiol Aging*. 2015;36(8):2444.e4. doi: 10.1016/j.neurobiolaging.2015.04.017 [doi].

16. Dhungel N, Eleuteri S, Li LB, et al. Parkinson's disease genes VPS35 and EIF4G1 interact genetically and converge on alpha-synuclein. *Neuron*. 2015;85(1):76-87. doi: S0896-6273(14)01081-2 [pii].

17. Wan H, Tang B, Liao X, Zeng Q, Zhang Z, Liao L. Analysis of neuronal phosphoproteome reveals PINK1 regulation of BAD function and cell death. *Cell Death Differ*. 2018;25(5):904-917. doi: 10.1038/s41418-017-0027-x [doi].

18. Kozlov G, Gehring K. Molecular basis of eRF3 recognition by the MLLE domain of poly(A)-binding protein. *PLoS One*. 2010;5(4):e10169. doi: 10.1371/journal.pone.0010169 [doi].

19. Pankratz N, Nichols WC, Uniacke SK, et al. Significant linkage of parkinson disease to chromosome 2q36-37. *Am J Hum Genet*. 2003;72(4):1053-1057. doi: S0002-9297(07)60629-6 [pii].

20. Pankratz N, Nichols WC, Uniacke SK, et al. Genome-wide linkage analysis and evidence of gene-by-gene interactions in a sample of 362 multiplex parkinson disease families. *Hum Mol Genet*. 2003;12(20):2599-2608. doi: 10.1093/hmg/ddg270 [doi].

21. Zimprich A, Schulte C, Reinthaler E, et al. PARK11 gene (GIGYF2) variants Asn56Ser and Asn457Thr are not pathogenic for parkinson's disease. *Parkinsonism Relat Disord*. 2009;15(7):532-534. doi: 10.1016/j.parkreldis.2009.01.005 [doi].

22. Schopp IM, Amaya Ramirez CC, Debeljak J, et al. Split-BioID a conditional proteomics approach to monitor the composition of spatiotemporally defined protein complexes. *Nat Commun*. 2017;8:15690. doi: 10.1038/ncomms15690 [doi].

23. Khurana V, Peng J, Chung CY, et al. Genome-scale networks link neurodegenerative disease genes to alpha-synuclein through specific molecular pathways. *Cell Syst*. 2017;4(2):170.e14. doi: S2405-4712(16)30445-8 [pii].

24. Joshi B, Cameron A, Jagus R. Characterization of mammalian eIF4E-family members. *Eur J Biochem*. 2004;271(11):2189-2203. doi: 10.1111/j.1432-1033.2004.04149.x [doi].

25. Morita M, Ler LW, Fabian MR, et al. A novel 4EHP-GIGYF2 translational repressor complex is essential for mammalian development. *Mol Cell Biol*. 2012;32(17):3585-3593. doi: 10.1128/MCB.00455-12 [doi].

26. Nishi K, Nishi A, Nagasawa T, Ui-Tei K. Human TNRC6A is an argonaute-navigator protein for microRNA-mediated gene silencing in the nucleus. *RNA*. 2013;19(1):17-35. doi: 10.1261/rna.034769.112 [doi].

27. Donato L, Bramanti P, Scimone C, Rinaldi C, D'Angelo R, Sidoti A. miRNAexpression profile of retinal pigment epithelial cells under oxidative stress conditions. *FEBS Open Bio*. 2018;8(2):219-233. doi: 10.1002/2211-5463.12360 [doi].

28. Cairns NJ, Grossman M, Arnold SE, et al. Clinical and neuropathologic variation in neuronal intermediate filament inclusion disease. *Neurology*. 2004;63(8):1376-1384. doi: 63/8/1376 [pii].

29. Cairns NJ, Uryu K, Bigio EH, et al. Alpha-internexin aggregates are abundant in neuronal intermediate filament inclusion disease (NIFID) but rare in other neurodegenerative diseases. *Acta Neuropathol*. 2004;108(3):213-223. doi: 10.1007/s00401-004-0882-7 [doi].

30. Gallart-Palau X, Serra A, Lee BST, Guo X, Sze SK. Brain ureido degenerative protein modifications are associated with neuroinflammation and proteinopathy in alzheimer's disease with cerebrovascular disease. *J Neuroinflammation*. 2017;14(1):y. doi: 10.1186/s12974-017-0946-y [doi].

31. Tramutola A, Di Domenico F, Barone E, et al. Polyubiquitinylation profile in down syndrome brain before and after the development of alzheimer neuropathology. *Antioxid Redox Signal*. 2017;26(7):280-298. doi: 10.1089/ars.2016.6686 [doi].

32. You NC, Chen BH, Song Y, et al. A prospective study of leukocyte telomere length and risk of type 2 diabetes in postmenopausal women. *Diabetes*. 2012;61(11):2998-3004. doi: 10.2337/db12-0241 [doi].

33. Liu D, O'Connor MS, Qin J, Songyang Z. Telosome, a mammalian telomere-associated complex formed by multiple telomeric proteins. *J Biol Chem*. 2004;279(49):51338-51342. doi: 10.1074/jbc.M409293200 [doi].

34. de Lange T. Shelterin: The protein complex that shapes and safeguards human telomeres. *Genes Dev*. 2005;19(18):2100-2110. doi: 19/18/2100 [pii].

35. Swanson MJ, Baribault ME, Israel JN, Bae NS. Telomere protein RAP1 levels are affected by cellular aging and oxidative stress. *Biomed Rep*. 2016;5(2):181-187. doi: 10.3892/br.2016.707 [doi].

36. Martinez P, Gomez-Lopez G, Pisano DG, Flores JM, Blasco MA. A genetic interaction between RAP1 and telomerase reveals an unanticipated role for RAP1 in telomere maintenance. *Aging Cell*. 2016;15(6):1113-1125. doi: 10.1111/acel.12517 [doi].

37. Forero DA, Gonzalez-Giraldo Y, Lopez-Quintero C, Castro-Vega LJ, Barreto GE, Perry G. Telomere length in parkinson's disease: A meta-analysis. *Exp Gerontol*. 2016;75:53-55. doi: 10.1016/j.exger.2016.01.002 [doi].

38. Cai Y, Sukhova GK, Wong HK, et al. Rap1 induces cytokine production in pro-inflammatory macrophages through NFkappaB signaling and is highly expressed in human atherosclerotic lesions. *Cell Cycle*. 2015;14(22):3580-3592. doi: 10.1080/15384101.2015.1100771 [doi].

39. Teo H, Ghosh S, Luesch H, et al. Telomere-independent Rap1 is an IKK adaptor and regulates NF-kappaB-dependent gene expression. *Nat Cell Biol*. 2010;12(8):758-767. doi: 10.1038/ncb2080 [doi].

40. Tan M, Wei C, Price CM. The telomeric protein Rap1 is conserved in vertebrates and is expressed from a bidirectional promoter positioned between the Rap1 and KARS genes. *Gene*. 2003;323:1-10. doi: S0378111903008795 [pii].

41. McMillan HJ, Humphreys P, Smith A, et al. Congenital visual impairment and progressive microcephaly due to lysyl-transfer ribonucleic acid (RNA) synthetase (KARS) mutations: The expanding phenotype of aminoacyl-transfer RNA synthetase mutations in human disease. *J Child Neurol*. 2015;30(8):1037-1043. doi: 10.1177/0883073814553272 [doi].

42. Seburn KL, Nangle LA, Cox GA, Schimmel P, Burgess RW. An active dominant mutation of glycyl-tRNA synthetase causes neuropathy in a charcot-marie-tooth 2D mouse model. *Neuron*. 2006;51(6):715-726. doi: S0896-6273(06)00673-8 [pii].

43. Verrigni D, Diodato D, Di Nottia M, et al. Novel mutations in KARS cause hypertrophic cardiomyopathy and combined mitochondrial respiratory chain defect. *Clin Genet*. 2017;91(6):918-923. doi: 10.1111/cge.12931 [doi].
